# Pairwise Additivity and Three-Body Contributions for Density Functional Theory-Based Protein–Ligand Interaction Energies

**DOI:** 10.1021/acs.jpcb.3c07456

**Published:** 2024-02-29

**Authors:** Charlotte Armida Elisabeth Schulze, Mauricio Cafiero

**Affiliations:** Department of Chemistry, University of Reading, Whiteknights, Reading RG6 6AP, U.K.

## Abstract

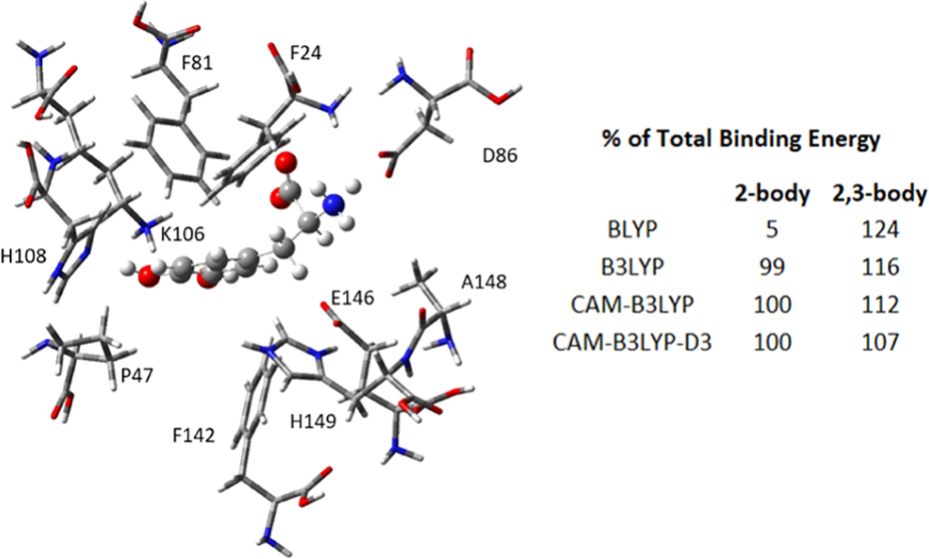

The prediction of protein–ligand binding energies is crucial in computer-assisted drug design. This property can be calculated in a straightforward fashion as the difference in the energies between a binding site–ligand complex and the separated binding site and ligand. Often, though, there is value in knowing how different amino acid residues in the protein binding site interact with the ligand. In this case, the interaction energy can be calculated as the sum of pairwise energies between each amino acid residue in the binding site and the ligand, and the sum of these energies is often equated with the total interaction energy. The validity of this pairwise additivity approximation can be assessed by experimental evidence, such as double-mutant cycles. In this work, we test the pairwise additivity approximation on the sulfotransferase-l-DOPA complex for 16 density functional theory (DFT) methods with varying degrees of exact (Hartree–Fock) exchange. Several “families” of functionals are studied, including BLYP, B3LYP, and CAM-B3LYP, as well as M06L, M06, and M062X. We also calculate the three-body contributions to interaction energy for the same DFT methods and assess when they are significant. We find that the amount of exact exchange or other nonlocal contributions has a direct influence on how closely the sum of pairwise energies approximates the total interaction energy. We also find that three-body interactions can be significant and that their significance can be predicted with good accuracy.

## 1 Introduction

When modeling the binding of a drug or drug-like ligand to a protein, the value most useful in comparison to experimental dissociation constant (*K*_d_) or inhibitory concentration (IC_50_) values is the free energy of binding. However, as Raha et al.^1^ have discussed, knowing how a specific fragment in the binding site interacts with a specific fragment on the ligand can aid in the drug design process. In that work, Raha and co-workers presented a decomposition of the ligand-binding site interaction into these pairwise interactions between ligand and binding site fragments. They showed that certain pairwise interactions correlate very well with the experimental Δ*G* of binding and that other pairwise interactions had no correlation. This demonstrates a use for the calculation of pairwise interactions in addition to total interactions in the process of drug design. In this work, pairwise as well as three- and four-body interactions for a specific example of ligand–protein binding (l-DOPA in the sulfotransferase, or SULT1A3, enzyme) will be studied and compared to total interaction energies. The SULT1A3 enzyme was chosen for this study for two reasons. First, it is an enzyme that is crucial to the life cycle of dopaminergic drugs, such as those used for treating Parkinson’s disease. One of the current authors has studied these types of molecules extensively,^2^ including in the SULT1A3 enzyme.^3^ Second, the binding site is of a reasonable size to perform three- and four-body calculations (10 amino acid residues, leading to 45 three-body energy terms) and contains an equal mixture of charged and polar amino acid residues (five) and nonpolar amino acid residues (five). This balance of residue types allows for the testing of the computational methods across a variety of intermolecular forces. The same ligand with a second enzyme, DOPA decarboxylase (DDC) was used to test the transferability of the results to a larger system. This enzyme is also crucial to Parkinson’s disease treatment and has been studied by the current author.^2^ The binding site of DDC is larger, with 13 residues, leading to 78 three-body interactions. Eighteen density functional theory methods are used in these calculations, and trends among different types of functionals are identified. Criteria for predicting important three-body interactions are presented. Although the focus of this work is on the additivity behavior of the density functional theory methods studied, and not on the absolute accuracy of the calculations presented, it is important to note that while the interaction energies calculated here are often strongly correlated to binding energies, they do not include zero-point energy/vibrational contributions, thermal or entropic contributions, or the contribution from desolvation/solvation during the drug binding process. This last point is often a very large contributor to binding energy and, in some cases, is the driving force in binding, so below we will briefly describe the solvation free energy of complexation and provide a computational example.

The idea that a total interaction energy can be decomposed into pairwise interactions between each protein residue and a ligand, called pairwise additivity, has been examined by experimental and computational studies. Wu and Prausnitz have shown that pairwise interactions are sufficient to describe the weaker, shorter-range forces involved in the interface of alkanes in water.^4^ These results are applicable to nonpolar amino acid residues and polar ligands. Using site-directed mutagenesis, a point mutation can be made in a protein binding site, altering one amino acid to another in order to evaluate the effect of that amino acid residue on the ligand-binding interaction. Often a strongly interacting residue will be mutated to alanine—a relatively weakly interacting residue, and the change in ligand binding will be attributed to that residue. Brix et al. have performed one such mutation experiment on the SULT1A3 enzyme, which is studied in the current work.^5^ They created mutants in which alanine, glutamine, and aspartate replaced a glutamate residue in the binding site, which changed the ligand-binding behavior dramatically. They then measured the Michaelis constant, *K*_m_, to measure binding affinity. The mutation to alanine showed an 8-fold increase in the value of *K*_m_ for the ligand dopamine, implying that the binding energy would grow weaker by about 16% (assuming  and 298.15 K). Dajani et al.^6^ also studied mutations in the SULT1A3 binding site, and they found that the mutation of the glutamate to alanine caused a decrease in the dopamine binding energy of 31%, double that of the work by Brix. These experimental results are supported by the previous work of one of the current authors, which shows that for the dopamine ligand, the glutamate residue contributes −88 kcal/mol to the total electronic interaction energy of −180 kcal/mol (M062*X*/6-311+G*).^2^

More rigorously, a double-mutant cycle can be created wherein two single-residue mutants and the corresponding double mutant are created, and the resulting ligand-binding energies for the wild-type and three mutants are measured. This allows for the estimation of the contributions of each individual residue to the overall binding and a measurement of cooperativity between the residues. Horovitz^7^ describes how double-mutant cycles can be used to measure Δ*G*_int_, which measures cooperativity, or how strongly the two residues interact. The work of Dajani et al.^6^ has such a double-mutant cycle for the SULT1A3 enzyme studied here, though they do not perform the Δ*G*_*int*_ analysis. As will be discussed below, their work shows that there is indeed strong cooperativity between residues in the SULT1A3 binding site. Due to this cooperativity, the pairwise additivity approximation should not hold for SULT1A3.

Medders et al. studied the pairwise additivity of calculated interactions in water clusters by calculating two-body and three-body interactions;^8^ if pairwise additivity holds, then three-body interactions should be negligible. The authors compared interactions calculated with several methods (including several of the density functional theory methods studied in the current work) with a reference of CCSD(T)/aug-cc-pVTZ. They found that global hybrid functionals and functionals with empirical dispersion energy corrections had better agreement with the two-body interaction reference values; this result is supported by the current work and is described below. The authors also found that all functionals did equally well in the three-body interaction accuracy. Their two-body interaction values clustered around −2 to −4 kcal/mol for all methods studied, and the three-body interaction values clustered around 0 kcal/mol for all methods but were not negligible. In their review, Cisneros et al. report that for water clusters, three-body interactions can be as high as 15–20% of the total interaction energy and that four-body interactions are typically close to 1%.^9^ Xantheas has reported three-body interactions in water clusters accounting for as much as 30% of total interaction energy.^10^

Ucisik et al. studied the total, two-, and three-body interaction energies of the ligand Indinavir to the protein HIV II protease using the DFT-based model chemistry M06*L*/6-31G*.^11^ They found that the sum of pairwise energies accounted for over 99% of the total interaction energy and that the magnitude of the sum of the three-body energies was about 4% of the sum of the two-body energies. Although that work used a basis set smaller than that used in the current work, those percentages align with that found in the current work.

The cost of three-body interaction calculations can be large compared to that of two-body interactions, so it would be helpful to predict which three-body interactions are non-negligible. The work of O’Flanagan et al.^12^ shows the importance of nearest neighbors in predicting large/important three-body interactions for DNA–protein binding using molecular mechanics calculations. This approach is evaluated for protein–ligand binding below and is found to be a good predictive tool here, as well. In the work presented below, the ratio of the sum of three-body interactions to two-body interactions is used as a measure of cooperativity in a system. The larger this fraction, the more important three-body interactions are to the system relative to two-body interactions.

## 2 Theory

Adapting the notation from the work of Ucisik et al.,^11^ the interaction energy of a ligand (l) in a protein binding site (bs) can be written as
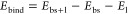
1The interaction energy can also be expressed as a sum of *n*-body terms, as derived by Xantheas^10^ and Ucisik et al.^11^ In this decomposition of the interaction energy, the system is broken down into 11 components: the 10 amino acid residues that comprise the binding site of SULT, and the ligand. We can define a two-body interaction energy, Δ^2^*E*(*i*,l), as
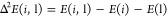
2where *E*(*i*,l) is the energy of the complex of the *i*-th residue with the ligand, *E*(*i*) is the energy of the *i*-th residue, and *E*(l) is the energy of the ligand. For our system, there are 10 two-body terms. Similarly, we can define a three-body interaction energy, Δ^3^*E*(*i*,*j*,l), as
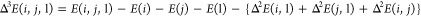
3where *E*(*i*,*j*,l) is the energy of the three-body complex of residues *i* and *j* and the ligand, and Δ^2^*E*(*i*,*j*) is the energy of the complex of residues *i* and *j*. The terms in the braces subtract the two-body energies from the total so that only the truly three-body effects remain. There are 45 three-body terms for our system, which require the calculation of 45 Δ^3^*E*(*i*,*j*,l) terms, as well as 45 Δ^2^*E*(*i*,*j*) that were not computed for the two-body energies. Four-body terms, Δ^4^*E*(*i*,*j*,*k*,l), are the highest-order terms studied in this work, and so we will define them here.
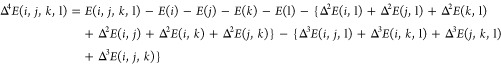
4Again, the terms in the brackets subtract the two- and three-body effects so that only true four-body effects remain. Five-body and higher terms can be derived similarly. Using these definitions, the decomposed interaction energy can be written as
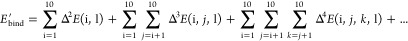
5In this work, we will explore the validity of truncating this interaction energy decomposition after the two- and three-body summations, and we will further explore the importance and magnitude of four-body terms.

In order to perform these calculations using Gaussian-orbital-based methods and DFT, each of the interaction energy calculations must be corrected for the basis set superposition error (BSSE). We perform the BSSE corrections using the counterpoise (CP) correction scheme.^13^ In this scheme, an interaction energy is calculated as in eq 1 or eq 2 using the same set of basis functions and DFT quadrature points for the whole complex as well as the individual components. This means that in a ligand residue complex, the complex calculation, the residue calculation, and the ligand calculation all have the same set of basis functions and DFT quadrature points. This set is usually defined to be that needed for the full complex calculation, meaning that the calculation for each component will have basis functions and quadrature points for the other component. When basis functions and quadrature points are centered around an atom that is not present in a particular step of a calculation (for example, around a residue atom when calculating the ligand energy), we say there is a ghost atom, which has no electrons or nuclear charge in that calculation, but which does have basis functions and quadrature points.

**Figure 1:**
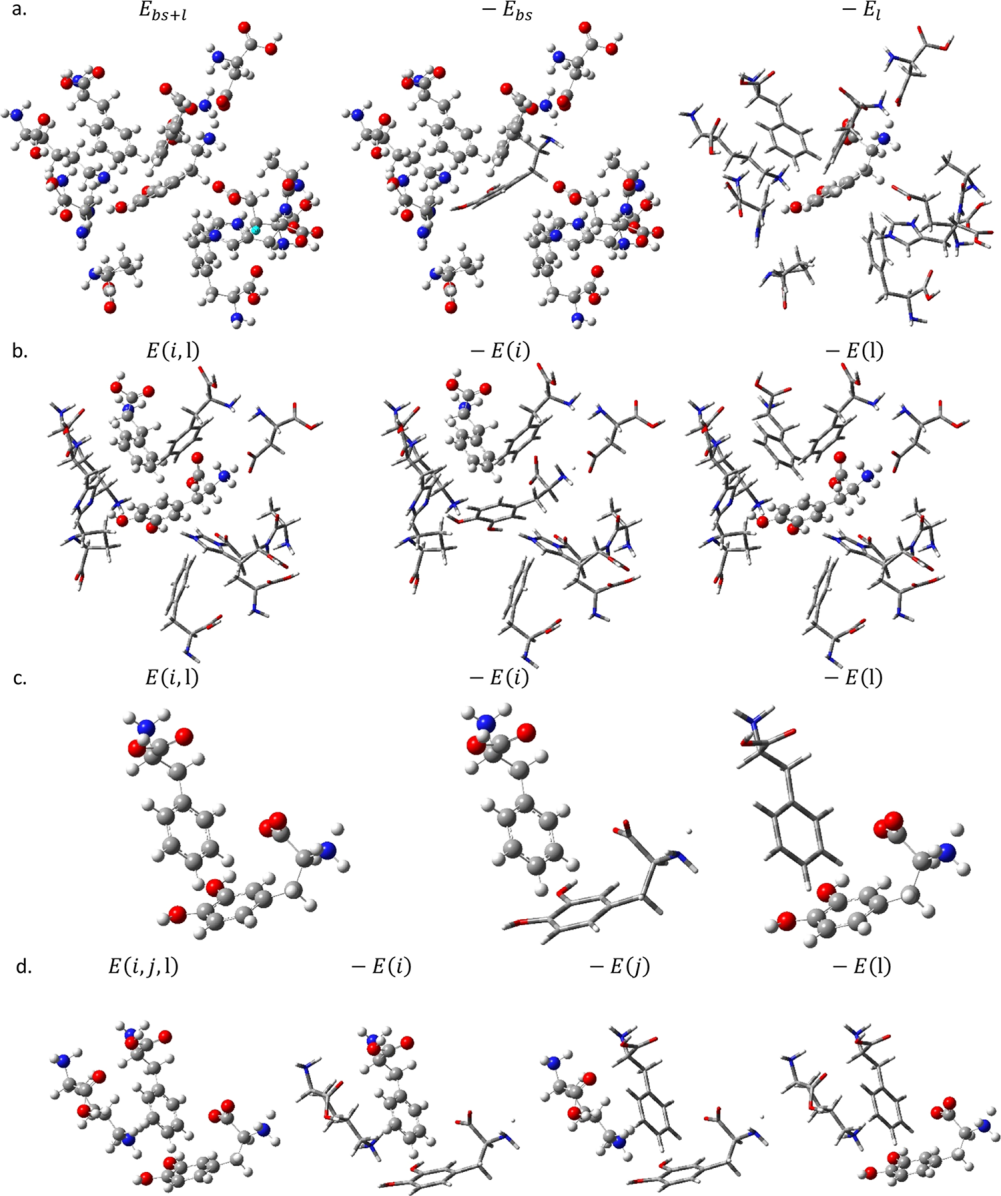
Example of counterpoise corrections for l-DOPA-Phenylalanine interactions. The atoms shown in the ball-and-stick theme are explicitly calculated, and the atoms in wireframe are treated as “ghost atoms” with basis functions and DFT grid points, but no electrons or nuclear charge: (a) counterpoise corrections for full interaction energy calculation (eq 1), (b) global counterpoise corrections for two-body l-DOPA-Phe81 energy, (c) local counterpoise corrections for two-body l-DOPA-Phe81 energy, (d) local counterpoise corrections for the three-body l-DOPA-Phe81-Lys106 energy.

For the calculations in eq 1, the CP correction scheme is simple: the set of basis functions and quadrature points for the binding site and ligand are used in all three energy calculations. For the *n*-body terms in eqs 2, 3, and 4, however, the choice of basis functions and quadrature points can be made to include the entire binding site+ligand complex (called global CP here) as done by Ucisik et al.,^11^ or it can be made to include only the basis functions and quadrature points for the *n*-bodies being studied (called local CP here). Figure 1 illustrates what ghost atoms are present in global and local CP calculations for two-body and three-body calculation.

## 3 Methods

**Figure 2:**
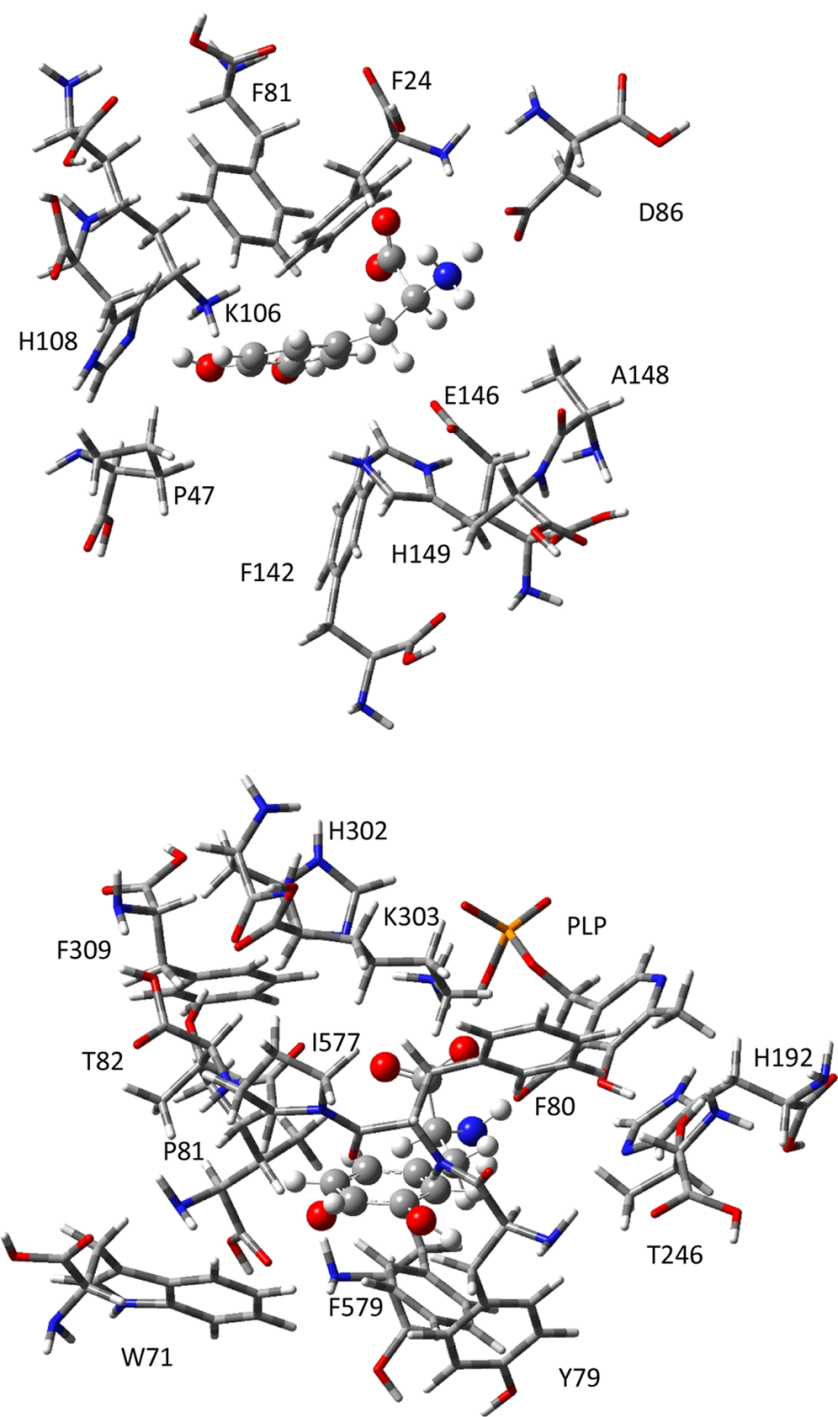
l-DOPA (ball-and-stick) in the SULT (top) active site (wireframe) and in the DDC active site (bottom), optimized with BMK/cc-pvdz and implicit solvation by water using the PCM.

The binding sites for the SULT1A3 and DDC enzymes were extracted from the crystal structures (PDB ID: 2A3R(14) for SULT1A3, 1JS3(15) for DDC) with the ligands dopamine and Carbidopa bound, respectively. The binding sites were defined as all amino acid residues with an atom within 3 Å of any atom of the bound ligand and included Ala148, Asp86, Glu146, His149, His108, Lys106, Phe142, Phe24, Phe81, and Pro47 for SULT1A3 and Phe579, Phe309, Phe80, Ile577, Trp71, Lys303, His192, His302, Pro81, Thr82, Thr246, Tyr79, and the cofactor pyridoxal phosphate (PLP) for DDC (see Figure 2). The cutoff of 3 Å for the binding site can be justified by the fact that most hydrogen bonds, dipole–dipole interactions, and ion–ion interactions in these systems have an atom–atom distance of less than 3 Å, and dispersion-type forces typically occur with a centroid-centroid distance of about 3 Å or less, so allowing residues with atom–atom distances of 3 Å or less covers those interactions as well. As will be shown in the results below, a cutoff of 3.4 Å was found to be needed for three-body interactions, so while there may be a situation where a residue outside of the 3 Å radius can pair with a residue inside the radius and influence the ligand, the interaction would be quite small, likely smaller than a typical in-radius three-body interaction energy of <|0.3| kcal/mol. All residues were capped with an −OH or an –H in order to maintain the physiological charge. In the full binding site structure with peptide bonds intact, the N-terminus of an amino acid is bonded to a C atom, so when truncating the N-terminus of a peptide bond, replacing the C on the connecting residue with a free H does not change the electron distribution of the N-terminus significantly, as the electronegativities of C and H are similar. However, when truncating the C-terminus, replacing the N on the connecting residue with a free H could significantly change the electron distribution as N and H have a greater difference in electronegativity, and H is less electronegative than C, rather than more electronegative like N. Thus, when truncating the C-terminus, the N is replaced with an OH, as the newly placed O and the N being replaced have a considerably smaller electronegativity difference than a free H and the N being replaced. In all cases of capping, the newly placed atoms are far enough away from the ligand that extra spurious interactions are negligible (i.e., they are at least 3 Å, and in many cases, farther), except for the cases of Tyr79 and Phe80 in the DDC binding site. In this case, the N–H group of the peptide bond between the two residues (which would be capped for Tyr79) binds to the ligand directly via hydrogen bonds. This interaction is captured in the pairwise Phe80-ligand calculation and in the three-body Tyr79-Phe80-ligand calculation, but for the Tyr79–ligand interaction, the broken bond is capped with an H rather than an OH so as to not double-count the interaction.

The bound ligands in each binding site were modified into l-DOPA, and the structures were optimized using BMK^16^/cc-pVDZ.^17,18^ In this optimization, the N–C_α_–C backbone of each residue was fixed in order to maintain the overall structure of the binding site from the crystal structure and all other atoms were allowed to relax. The optimizations included solvation by water using the polarizable continuum model^19^ (PCM). The ligand was initially built in a zwitterionic state in both binding sites, as dictated by the p*K*_a_ of the amine and carboxyl groups at physiological pH. In the case of SULT1A3, the ligand retained both charges after optimization, whereas in the case of DDC, a proton from the –NH_3_^+^ group transferred to the charged PLP cofactor. Charged amino acid residues were prepared in their charge state at physiological pH, unless shown to have a different charge state in the crystal structure. Previous work by one of the authors has shown how optimization in the presence of implicit solvent allows for ion stabilization compared to optimizations *in vacuo*.^3^ These optimized structures were used for all subsequent calculations.

Several “families” of DFT methods were used in this study: HCTH,^20^ τHCTH,^21^ and τHCTHhyb,^21^ along with the related BMK functional;^16^ BLYP,^22,23^ B3LYP,^24^ CAM-B3LYP,^25^ and the empirical dispersion-corrected CAM-B3LYP-D3;^26^ M06L,^27^ M06,^28^ M062X,^28^ and the empirical dispersion-corrected M062X-D3,^26^ along with the related MN12SX functional;^29^ and PBE,^30^ PBE1PBE,^31^ LC-wHPBE,^32^ and the related TPSS functional.^33^ The SVWN functional^34,35^ and the Hartree–Fock (HF) method were also tested for comparison. All energy calculations were performed with the aug-cc-pVDZ basis set,^17,18^ except the M06L calculations which were performed for comparison with the work of Ukisik et al.,^11^ which were run with the 6-31G* basis set.^36^ All calculations included the PCM implicit solvation with water.

### 3.1 Total Interaction Energy Calculations

The total interaction energy calculations (eq 1) were performed with the 18 DFT methods described above and the HF method. CP corrections were applied as in Figure 1a, wherein the energy calculations for the binding site included ghost atoms from the ligand and the energy calculation for the ligand included ghost atoms from the entire binding site.

### 3.2 Two-Body Energy Calculations

The two-body energy calculations (eq 2) were performed with the 18 DFT methods described above and the HF method. Global CP corrections were applied as in Figure 1b for three sample series (BMK/aug-cc-pVDZ, tHCTH/aug-cc-pVDZ, and M06*L*/6-31G*), wherein energy calculations for the ligand included ghost atoms from the entire binding site and energy calculations for each amino acid residue included ghost atoms on the ligand and the other 9 residues. Local CP corrections were applied as in Figure 1c for all 18 DFT methods and HF, wherein energy calculations for the ligand included ghost atoms from the *i*-th amino acid residue only, and energy calculations for the *i*-th amino acid residue included ghost atoms on the ligand only. Ten body terms were calculated per the DFT method.

### 3.3 Three-Body Energy Calculations

The three-body energy calculations (eq 3) were performed with the 18 DFT methods described above and the HF method. Local CP corrections were applied as in Figure 1d for all DFT methods, wherein energy calculations for the ligand included ghost atoms from the *i*-th and *j*-th amino acid residues, the energy calculations for the *i*-th amino acid residue included ghost atoms on the ligand and *j*-th residue, and the energy calculations for the *j*-th amino acid residue included ghost atoms on the ligand and *i*-th residue. Global CP was not attempted on three-body interactions, and the magnitude of CP corrections for three-body interactions is generally smaller than that for two-body interactions, as has been reported.^8^ The three-body calculations included 45 three-body energies and 45 two-body energies (none including the ligand) per the DFT method.

### 3.4 Four-Body Energy Calculations

The four-body energy calculations (eq 3) were performed with BMK/aug-cc-pVDZ. Local CP corrections were applied in analogy to the three-body calculations shown in Figure 1d, wherein energy calculations for the ligand included ghost atoms from the *i*-th, *j*-th and *k*-th amino acid residues, the energy calculations for the *i*-th amino acid residue included ghost atoms on the ligand and *j*-th and *k*-th residues, the energy calculations for the *j*-th amino acid residue included ghost atoms on the ligand and *i*-th and *k*-th residues, and the energy calculations for the *k*-th amino acid residue included ghost atoms on the ligand and *i*-th and *j*-th residues. Only two sample four-body terms have been calculated as examples.

### 3.5 Solvation Free Energy of Complexation

The solvation energy of complexation was calculated for one sample method/basis set, BMK/aug-cc-pVDZ in order to provide relative magnitudes of the solvation energy and the interaction energy. The ΔΔ*G*_solv_ was calculated according to the following cycle presented by Raha et al.^1^
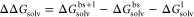
6where each component was calculated with BMK/aug-cc-pVDZ and implicit solvation via the PCM. The same optimized binding site/ligand complex used above was used here.

All calculations above were performed with the Gaussian 16 software.^37^

## 4 Results and Discussion

Raw data for all of the results can be found in Tables S1–S5.

### 4.1 Global and Local Counterpoise Corrections

Three model chemistries were chosen to study the effects of global and local CP corrections: a pure meta-GGA DFT method (τHCTH) and a global hybrid method (BMK) both with the aug-cc-pVDZ basis set and the pure meta-GGA M06L method with 6-31G*. The first two methods were chosen to test the effect of HF exchange on the CP corrections, and the third method and basis set were chosen in order to compare with the work of Ucisik et al.^11^Table 1 shows individual two-body energy terms between the ligand l-DOPA and each amino acid residue in the SULT1A3 binding site for each of the three methods with three levels of CP correction. For each method, it can be seen that there is a large difference between no CP and local CP for each two-body term: about 1 kcal/mol on average for the two methods using aug-cc-pVDZ and about 0.15 kcal/mol on average for the method using 6-31G*. Table 1 also shows the sum of the two-body terms, with a difference between the totals of about 10 kcal/mol (aug-cc-pVDZ) or 1.5 kcal/mol (6-31G*). Looking at the last column of Table 1, the timings (total core time) for a l-DOPA/Phe142 calculation are given. For τHCTH/aug-cc-pVDZ, the time is roughly doubled between no CP and local CP, but for the BMK/aug-cc-pVDZ method with HF exchange, the time increase is about 4-fold. This comes from roughly doubling the amount of integration points and basis functions in the two fragment calculations in the CP correction.

**Table 1:** Comparison of Global Counterpoise Corrections to Basis Set Superposition Errors (BSSE) to “Local” Counterpoise Corrections to BSSE and No Counterpoise Corrections for BSSE for a GGA, Meta-GGA, and Global Hybrid Meta-GGA DFT Method^a^

	Δ^2^*E_i_*											
method	A148	D86	E146	H149	H108	K106	F142	F24	F81	P47	sum Δ^2^*E_i_*	*t*(ld-Phe142)
BMK, no CP	0.09	–12.67	–0.24	0.83	–6.21	–4.31	0.26	–0.45	0.47	0.20	–22.02	295
BMK, local CP	0.33	–16.50	–4.06	1.11	–7.44	–7.27	0.98	–0.16	0.03	0.44	–32.54	1168
BMK, global CP	0.37	–16.52	–5.11	1.48	–7.43	–7.19	1.03	–0.62	0.17	0.44	–33.39	43 571
tHCTH, no CP	–0.40	–13.87	2.37	–0.24	–6.65	–3.37	–0.57	3.01	5.46	–0.45	–14.70	203
tHCTH, local CP	–0.27	–17.52	–1.50	–0.09	–7.87	–6.15	0.00	3.03	4.92	–0.28	–25.73	406
tHCTH, global CP	–0.23	–17.43	–2.52	0.21	–7.82	–6.05	0.02	2.58	5.01	–0.30	–26.52	21 435
M06L, no CP	–0.22	–19.21	–5.92	–0.12	–8.37	–8.19	–1.07	–4.71	–4.04	–0.29	–52.14	28
M06L, local CP	–0.19	–20.17	–6.15	–0.12	–8.93	–10.34	–0.72	–3.35	–3.60	–0.20	–53.77	44
M06L, global CP	–0.15	–20.35	–6.69	0.17	–8.68	–10.13	–0.71	–3.76	–3.53	–0.23	–54.06	731

a Basis set is aug-cc-pVDZ for BMK and τHCTH and 6-31G* for M06L. The final column is the core time for a full interaction energy calculation between l-DOPA and Phe142. Energy values are given in kcal/mol and time in minutes.

In going from the local CP corrections to the global CP corrections, the difference in two-body energy terms is quite small compared to the no CP/local CP difference: about 0.1 kcal/mol (aug-cc-pVDZ) or 0.03 kcal/mol (6-31G*). The time increase in going from local CP to global CP is large: a 50-fold increase in time for τHCTH, a 37-fold increase in time for BMK, and a 17-fold increase in time for M06L/6-31G*. Thus, due to the small change in energy values and the large increase in computing time, only local CP will be used for the rest of this work.

### 4.2 Pairwise Additivity and Exact Exchange for SULT1A3

Table 2 shows the interaction (or electronic binding) energy between l-DOPA and the SULT1A3 binding site calculated in three ways: the total energy (eq 1), the sum of two-body terms, and the sum of two- and three-body terms. Also presented are several ratios: the sum of all three-body terms to two-body terms, the sum of two-body terms to the total energy, and the sum of two- and three-body terms to the total. The DFT methods are arranged in order of increasing nonlocality, with local or GGA and meta-GGA methods first, followed by global hybrid or range-separated hybrid methods, and then followed by methods using empirical dispersion.

**Table 2:** Ligand–Protein Interaction Energies (IEs) Calculated in Three Ways with 18 DFT Methods and the aug-cc-pVDZ Basis Set Unless Otherwise Indicated^a^

method	IE tot	IE 2B	3B	IE 2B + 3B	(3B/IE 2B) × 100	(IE 2B/IE tot) × 100	(IE 2B+3B/IE tot) × 100
HCTH	–17.64	–23.60	2.23	–21.37	9	134	121
tHCTH	–22.38	–25.73	–0.47	–26.20	2	115	117
τHCTHhyb	–33.65	–34.36	–3.31	–37.67	10	102	112
BMK	–38.04	–32.54	–9.98	–42.52	31	86	112
M06L	–56.11	–52.02	–8.65	–60.67	17	93	108
M06	–55.24	–51.49	–8.03	–59.52	16	93	108
M062X	–57.24	–55.92	–5.38	–61.30	10	98	107
M062X-D3	–63.43	–62.13	–5.35	–67.48	9	98	106
MN12SX	–47.75	–44.57	–7.20	–51.77	16	93	108
BLYP	–18.40	–17.48	–5.28	–22.75	30	95	124
B3LYP	–26.68	–26.34	–4.60	–30.94	17	99	116
CAM-B3LYP	–37.09	–37.18	–4.17	–41.36	11	100	112
CAM-B3LYP-D3	–60.84	–60.98	–4.12	–65.10	7	100	107
PBE	–35.34	–37.14	–2.17	–39.31	6	105	111
PBE1PBE	–37.61	–38.58	–3.06	–41.63	8	103	111
LC-ωHPBE	–35.36	–34.36	–5.19	–39.55	15	97	112
TPSS	–28.51	–29.50	–2.91	–32.41	10	103	114
SVWN	–75.58	–78.26	–1.47	–79.73	2	104	105
HF	–15.96	–15.45	–4.71	–20.17	31	97	126
M06*L*/6-31G*	–56.97	–53.77	–6.53	–60.30	12	94	106

a IE tot = *E*_P + L_ – *E*_P_ – *E*_L_; IE 2B = ∑Δ^2^*E_i_*; 3B = ∑∑Δ^3^*E*_*ij*_; IE 2B + 3B = ∑Δ^2^*E*_*i*_ + ∑∑Δ^3^*E*_*ij*_. All values in kcal/mol.

**Figure 3:**
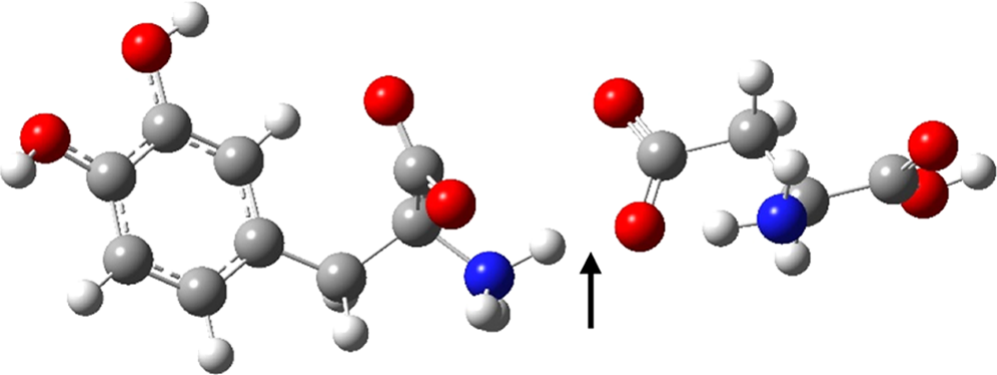
l-DOPA-Asp86 complex, with the middle point between the N–H and O^–^ identified.

**Figure 4:**
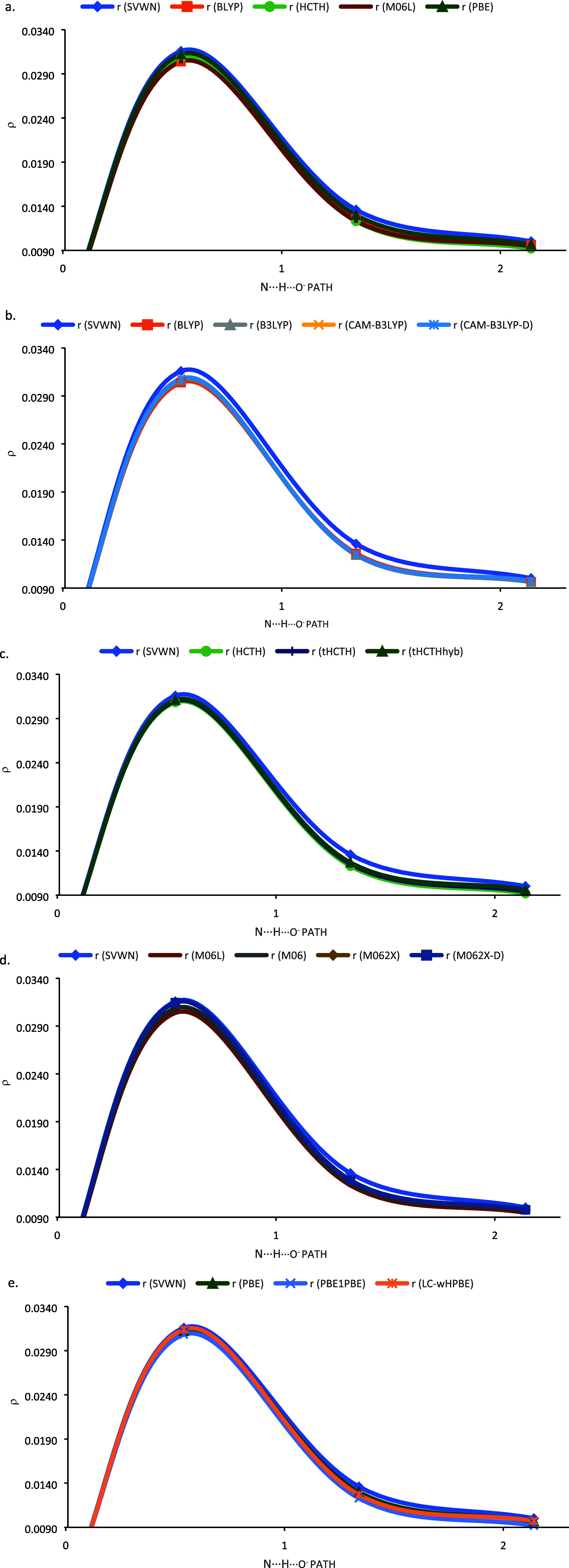
Electron density along the hydrogen-bond path from the N–H to the O^–^ in the l-DOPA-Asp86 complex, shown in Figure 3. The origin is the N nucleus, the first point is the H nucleus, the second point is the midpoint between the H and the O nuclei, and the last point is the O nucleus. (a) SVWN compared BLYP, HCTH, M06L, and PBE; (b) SVWN compared with the BLYP family of functionals; (c) SVWN compared with the HCTH family of functionals; (d) SVWN compared with the M06L family of functionals; (e) SVWN compared with the PBE family of functionals. Basis set is aug-cc-pVDZ in all cases.

SVWN has the strongest total interaction energy of the methods studied at −75.58 kcal/mol. This is due to overestimating the electron density between separated molecules. Figure 3 shows the complex of Asp86 and the l-DOPA ligand and indicates the center of a hydrogen bond between the N–H on l-DOPA and the O^–^ on Asp86. Figure 4a shows the electron density plotted on a path from N–H to the O^–^ with SVWN and four other functionals. It can be seen that in comparison to the other functionals in Figure 4, SVWN has a noticeably higher density at both the H nucleus and the center of the hydrogen bond. HF has the weakest total interaction energy studied at −15.96 kcal/mol. All other DFT methods fall between these. The GGA methods BLYP and HCTH have the smallest magnitude of interaction energy at around −18 kcal/mol due to how they correct the SVWN/LSDA electron density. BLYP and HCTH have considerably lower electron densities along the h-bond, as can be seen in Figure 4. The meta-GGA and hybrid methods’ nonlocality fixes the under and overcorrection of the GGA methods and generally increases the magnitude of the total interaction energy (Table 2). Although the trends are different for each family of functionals, the density points in Figure 4b–e illustrate how the addition of nonlocality to the functional affects the nonbonded electron density in the h-bond example. Further, the Minnesota functionals have an overall stronger interaction energy than the HCTH-, BLYP-, and PBE-based methods, although the empirical correction to CAM-B3LYP brings it close to the values calculated by the Minnesota methods.

In order to obtain the total interaction energies for a ligand-binding site complex, solvation-desolvation of the ligand and binding site must be taken into account. Although these solvation calculations are beyond the scope of this work, we have investigated the solvation energy of complexation for one model chemistry, BMK/aug-cc-pVDZ. The total interaction energy (Table 2) for this method is −38.04 kcal/mol. The solvation energy of complexation, calculated via eq 6, is +48.47 kcal/mol, with a total interaction energy + solvation energy of complexation of +10.44 kcal/mol.

The third column of Table 2 shows the interaction energy as calculated by eq 5 truncated after the two-body sum. This energy represents the pairwise additivity approximation or the idea that the sum of the pairwise energies should approach the total energy of eq 1. The seventh column of Table 2 shows the percentage of the sum of the pairwise energies (labeled IE 2B) to the total energy; a value of 100% for this ratio would mean that the sum of the two-body terms exactly equals the total energy. As each series of DFT methods progresses from less nonlocality to more nonlocality, the ratio approaches 100, although from different directions. For the series HCTH → τHCTH → τHCTHhyb, the ratio goes from 134% to 115% to 102%. On the other hand, for the series BLYP → B3LYP → CAM-B3LYP → CAM-B3LYP-D3, the ratio goes from 95% to 99% to 100% to 100%. It can be said that as a DFT method is corrected for nonlocality, the more pairwise additivity holds true, as the most-nonlocal functional of each family has the ratio closest to 100%. This is due to the nonlocal elements (HF exchange, kinetic energy density, or empirical dispersion) being better able to model long-range forces that would otherwise be lost with only pairwise interactions. The third column of Table 3 shows the percentage averaged for types of DFT methods, including GGA, meta-GGA, global hybrid, range-separated, and empirical dispersion. It can be seen that, when averaged over all DFT methods studied, the sum of two-body interactions accounts for 101% of the total interaction energy, suggesting that, in general, pairwise additivity holds for DFT-based approaches to this ligand–protein binding.

**Table 3:** Average Values Grouped by Type of DFT Method^a^

	(3B/IE 2B) × 100	(IE 2B/IE total) × 100	(IE 2B + 3B/IE total) × 100
average	13	101	112
average GGA	15	111	119
average MGGA	9	104	113
average GGA-H	13	101	113
average MGGA-H	16	95	110
average RS	14	97	111
average + D	8	99	107

a IE 2B = ∑Δ^2^*E*_*i*_; 3B = ∑∑Δ^3^*E*_*ij*_; and IE 2B + 3B = ∑Δ^2^*E*_*i*_ + ∑∑Δ^3^*E*_*ij*_. All values in kcal/mol.

Examining the trends of Table 3 by DFT type, however, shows that the only type of DFT method that averages to 101% by itself is GGA-hybrid, although the empirical dispersion-corrected methods average to 99%. GGA methods average 111%, suggesting that the under and overcorrection in GGA compared to SVWN is more pronounced in total interaction energy calculations than in the pairwise calculations. This would be due to the effect of the density gradient being more pronounced in a larger system than in a smaller system, i.e., more of the long-range behavior is lost in the larger systems. Meta-GGA and hybrid-GGA relax that under and overcorrection and bring the percentage down to 104% and 101% as they improve the description of long-range behavior, and hybrid meta-GGA and range-separated methods bring it down even further to 95 and 97%, respectively. These last two percentages may be the more accurate representation of the pairwise additivity of DFT, as they offer the best description of long-range forces that come into play in many-body interactions. Ucisik et al. reported that the pairwise interactions in the ligand–protein system they studied using M06L/6-31G* accounted for 99% of the total interaction energy.^11^ The fact that this is higher than the current estimate is attributable to several reasons. First, the systems are different, and M06*L*/6-31G* applied to the system in this study gives a percentage of 94%. Second, the basis set used in this study is larger than the one used by Ucisik, and so more of the long-range behavior is captured in the total interaction energy calculation, making the denominator in the ratio larger and decreasing the percentage.

### 4.3 Three and Four-Body Interactions for SULT1A3: Importance and Magnitude

The fourth column of Table 2 shows the sum of the three-body interactions for all of the DFT methods studied here. Almost all are attractive interactions (−) which would increase the total interaction energy, except for HCTH, which has a repulsive value for this total (+), meaning that it would decrease the overall interaction energy. The sixth column of Table 2 shows the ratio of the sum of the three-body interactions to the sum of the two-body interactions expressed as a percentage. It can be seen that the three-body interactions can be anywhere from 2 to 31% of the two-body interactions. For the M06L and BLYP-based families of functionals, this percentage decreases when more nonlocality is added to the method. For BLYP → B3LYP → CAM-B3LYP → CAM-B3LYP-D3, the percentage goes from 30% to 17% to 11% to 7%. In BLYP, the two-body interactions are an underestimate of the total interaction energy at 94%, and so, the ratio is higher than it would be otherwise. Going to B3LYP, the two-body interactions increase to 99% of the total interaction energy, so the three-body interaction ratio is smaller. Going further to CAM-B3LYP, the two-body interactions are 100% of the total interaction energy, and so the three-body interaction ratio decreases again. At the same time, the magnitude of the sum of the three-body interactions decreases along this series. This is because as nonlocality increases, the under-correction of SVWN/LSDA is addressed (as may be seen in Figure 4), decreasing the magnitude of the three-body energies. This same trend can be seen for the M06L-based family. In this case, the percentage of three-body interactions to two-body interactions decreases from 17 to 16% to 10 to 9% as the series goes from M06L → M06 → M062X → M062X-D3.

The other two families of functionals (HCTH and PBE-based) do not show this same behavior. In both of those families, the attractiveness of the three-body interactions grows larger as more nonlocality is added, rather than decreasing as in the other two series. At the same time, the two-body interactions are larger than the total interaction energy, rather than smaller as with the other two series, though they do converge toward 100% as more nonlocality is added. Going from HCTH → τHCTH → τHCTHhyb, the two-body interactions account for 134, 115 and 102% of the total interaction energy. Likewise, in the series PBE → PBE1PBE → LC-ωHPBE, the two-body interactions account for 105, 103, and 97% of the total interaction energy. This is due to the fact that these functionals, unlike the BLYP-based series, *overcorrect* the SVWN density (rather than *under-correct*) and thus the percentage of three-body interactions compared to two-body interactions *increases* as more nonlocality is added.

The large percentages of three-body interactions to two-body interactions show that pairwise additivity does not hold in this example system. The second column of Table 3 shows the average of the percentage of three-body interactions to two-body interactions, and over all DFT methods, this average is 13%, with the smallest average (for the method with empirical dispersion) being 8%. Such large percentages indicate that three-body interactions cannot be neglected in this system. This fact is emphasized by the double-mutant cycle experiment of Dajani et al.^6^ In this experiment, the authors created mutants by changing the Glu146 residue to alanine and the His143 residue to tyrosine and created a double mutant by changing both together. The binding energies of dopamine and 4-nitrophenol were measured for all four proteins. By extracting the measured *K*_m_ values from that work and converting them to Δ*G*_bind_, the Δ*G*_int_ values can be calculated. For dopamine and 4-nitrophenol binding, the Δ*G*_int_ values were −0.78 and 0.22, respectively. Both values show that considerable cooperativity exists between these residues, with the larger negative value for dopamine because dopamine extends to the part of the binding site that contains those two residues and interacts with them attractively, and 4-nitrophenol does not.

**Figure 5:**
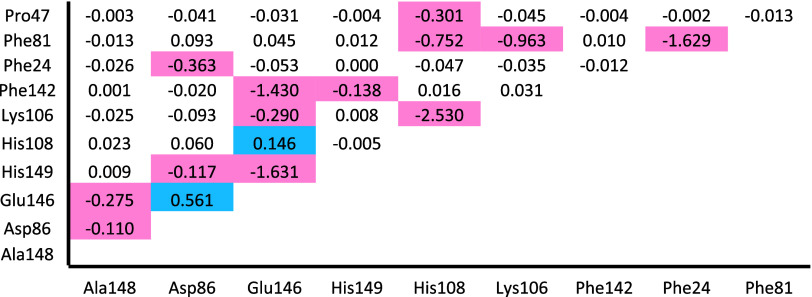
Matrix of three-body interaction energies (kcal/mol) between l-DOPA and each pair of residues in the SULT1A3 binding site calculated with BMK/aug-cc-pVDZ and implicit solvation by water. Values highlighted in red are less than −0.1, and values highlighted in blue are greater than 0.1.

Figure 5 shows all of the three-body interactions calculated with BMK for this system, highlighting the values greater than |0.1 kcal/mol|. As can be seen in Table 2, these three-body interactions total −9.98 kcal/mol. The three largest three-body interactions are for l-DOPA with Glu146/His149, with His108/Lys106, and with Phe24/Phe81. The large, negative value for Glu146/His149 agrees with the double-mutant cycle analysis above. The three-body interactions are costly to calculate, as they require 315 separate calculations for this system (including CP corrections). Two-body interactions only require 30 calculations, and take less memory and core time than the three-body calculations.

**Figure 6:**
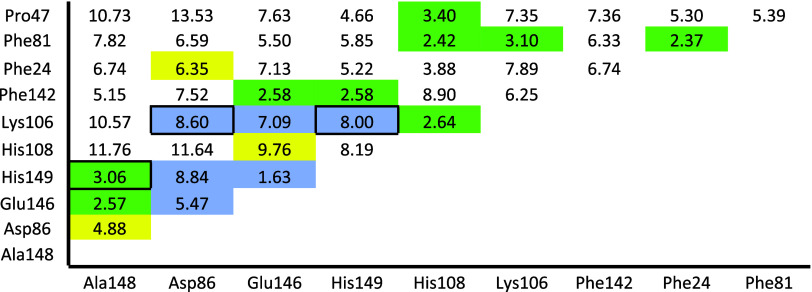
Matrix of inter-residue distances (Å) in the SULT1A3 binding site (optimized in the presence of l-DOPA using BMK/aug-cc-pVDZ and implicit solvation by water) based on closest side-chain atoms. Values highlighted in green indicate residues that are closer than 3.4 Å; values highlighted in blue indicate residues that can interact via charge–charge interactions; values highlighted in yellow indicate residue pairs that have significant three-body energies with l-DOPA but are not captured by the previous two criteria; values in a black border indicate residue pairs that meet the previous two criteria but do not have significant three-body interactions with l-DOPA.

The work of O’Flanagan et al. suggests nearest neighbors as a criterion for cooperativity in DNA–protein binding. Figure 6 shows the inter-residue distances in the SULT1A3 binding site based on the closest side-chain atoms for each residue pair. If the calculation of three-body interactions is limited to only those residues close enough to have a strong interaction, considerable time can be saved. An inter-residue distance of 3.4 Å was chosen as a criterion for choosing which residue pairs to include in the three-body interactions, as that is the distance of a typical ring–ring interaction, which is one of the weaker interactions found in the binding site. In addition, all residue pairs that can interact via charge–charge interactions were included for three-body interactions. If all of the 15 residue pairs that meet these criteria are added, a total three-body energy of −9.57 kcal/mol is obtained, compared to −9.98 for all 45 residue pairs. Thus, 96% of the three-body energy is recovered with only one-third of the computational time and expense. It should also be noted that the three residue pairs with the largest three-body interactions (Glu146/His149, His108/Lys106, and Phe24/Phe81) are also the three with the smallest inter-residue distances, indicating that this criteria is valid.

Two four-body interaction terms were calculated to test the potential magnitudes of these terms. The His108–Lys106–Phe81 interaction with l-DOPA was the first four-body term examined. As can be seen in Figure 6, His108 and Lys106 are among the closest residues at 2.64 Å and have a large three-body interaction with l-DOPA. Likewise, Lys106 and Phe81 are within the proximity criteria at 3.10 Å, and His108 and Phe81 are even closer at 2.42 Å. According to the three-body prediction scheme of nearest neighbors, this four-body interaction should be one of the more significant terms. The four-body interaction energy, calculated as in eq 4, was 0.03 kcal/mol. A second four-body term was calculated for the Lys106/Phe24/Phe81 interaction with l-DOPA. In this case, Lys106 is close to Phe81 (3.10 Å) and Phe24 is close to Phe81 (2.37 Å), but Lys 106 is not close to Phe24, so this interaction should be less than the His108/Lys106/Phe81 term. The calculated four-body interaction for this second cluster is also 0.03 kcal/mol. The fact that these two are of the same magnitude and sign and that they are 2 orders of magnitude smaller than the more significant three-body interactions suggests that the contribution of the four-body terms may not be significant.

### 4.4 Transferability of Results to DDC

The total, two-body, and three-body calculations were repeated with the optimized l-DOPA/DDC complex to test the transferability of the results. The DDC binding site contains 13 residues (including the PLP cofactor) and so has 78 three-body terms and 78 associated two-body terms that do not include the ligand. DDC was thus studied with one “family” of DFT methods: BLYP, B3LYP, CAM-B3LYP and CAM-B3LYP-D3. This set was chosen due to the popularity of the B3LYP-based functionals and due to the fact that the two-body and three-body convergence behavior of this set for SULT1A3 was very clear. Table 4 shows the total, two-body, and three-body energies as well as some percentages for both the SULT1A3 and DDC/l-DOPA complexes for the B3LYP set of functionals.

**Table 4:** Ligand–Protein Interaction Energies (IEs) Calculated in Three Ways with Four DFT Methods and the aug-cc-pVDZ Basis Set^a^

method	IE tot	IE 2B	3B	IE 2B + 3B	(3B/IE 2B) × 100	(IE 2B/IE tot) × 100	(IE 2B + 3B/IE tot) × 100
SULT1A3							
BLYP	–18.40	–17.48	–5.28	–22.75	30	95	124
B3LYP	–26.68	–26.34	–4.60	–30.94	17	99	116
CAM-B3LYP	–37.09	–37.18	–4.17	–41.36	11	100	112
CAM-B3LYP-D3	–60.84	–60.98	–4.12	–65.10	7	100	107
DDC							
BLYP	–13.22	–9.68	–4.88	–14.56	50	73	110
B3LYP	–23.22	–20.33	–3.73	–24.05	18	88	104
CAM-B3LYP	–35.68	–33.39	–2.91	–36.31	9	94	102
CAM-B3LYP-D3	–64.78	–63.22	–2.18	–65.41	3	98	101

a IE tot = *E*_P + L_ – *E*_P_ – *E*_L_; IE 2B = ∑Δ^2^*E*_*i*_; 3B = ∑∑Δ^3^*E*_*ij*_; IE 2B + 3B = ∑Δ^2^*E*_*i*_ + ∑∑Δ^3^*E*_*ij*_. All values in kcal/mol.

The total energies for the l-DOPA/DDC complex follow the same trend as in SULT1A3, with the attraction increasing as more nonlocality is added to the functional. The sum of the two-body energies follows the same trend, with the sum of the two-body energies (IE 2B) closely approximating the total energy. The ratio of two-body energies to the total increases along the series from 73% for BLYP to 98% for CAM-B3LYP-D3, as the more nonlocal functionals can replicate the longer-range interactions in the total energy calculations. The trend for DDC is very similar to that found for SULT1A3 and shows that from B3LYP onward, pairwise additivity holds for this system.

The DDC three-body energies also follow the same trends as those of SULT1A3. The ratio of the sum of three-body energies to the sum of two-body energies decreases from 50 to 3% along the series, showing a slightly better convergence than the SULT1A3 results. The ratio of the sum of the DDC two-body and three-body energies to the total energy also shows excellent convergence, going from 110% for BLYP to 101% for CAM-B3LYP-D3. Again, this is a better convergence than that seen in the SULT1A3 results.

Overall, the trends for the l-DOPA/DDC complex follow the trends shown for the SULT1A3 complex and even have slightly better convergence behavior in both pairwise additivity and many-body additivity.

## 5 Conclusions

Pairwise additivity for DFT methods is a valid approximation for methods with some added nonlocality beyond the GGA, such as global hybrid functionals, meta-GGA functionals, or methods with added empirical dispersion; this has been shown in two ligand/enzyme binding site systems. Taking the ratio of the sum of pairwise interactions to the total interaction energy from the hybrid meta-GGA and range-separated methods as the most accurate measure of the pairwise additivity, it can be concluded that pairwise interactions account for about 96% of the total interaction energy for ligand–protein systems such as the one in this study, calculated with DFT.

Three-body interactions as calculated by DFT methods can be significant, with the ratio of three-body interactions to two-body interactions being around 13% for all of the methods studied. Calculation of the three-body terms can be costly, but using nearest neighbors and charge–charge interactions between residues as a selection criteria, the main contributors to three-body interactions can be predicted, and in this work, the use of predicted significant three-body terms accounts for 96% of the three-body energy and saves 67% of computing time and expense. Four-body interactions are considerably more costly than three-body interactions. Some sample four-body energies calculated here, which were predicted to be among the more significant contributors to the energy, were 2 orders of magnitude smaller than the significant three-body terms. Thus, for systems like those considered in this work, four-body interactions can be safely ignored.

When calculating total interaction energies, global CP corrections should be used, but when calculating pairwise interactions, local CP corrections account for 97% of the pairwise energies at around 2–3% of the computational time. As the magnitude of the CP corrections decreases with higher-order n-body interactions, only local CP corrections are suggested for all many-body calculations.

## Supporting Information Available

Raw data for total, pairwise, three-body, and four-body interaction energies for each DFT method; raw data for CP study (XLSX)
